# Chromosome-level genome assembly of the spotted alfalfa aphid *Therioaphis trifolii*

**DOI:** 10.1038/s41597-023-02179-y

**Published:** 2023-05-12

**Authors:** Tianyu Huang, Yang Liu, Kang He, Frédéric Francis, Bing Wang, Guirong Wang

**Affiliations:** 1 State Key Laboratory for Biology of Plant Diseases and Insect Pests, Institute of Plant Protection, Chinese Academy of Agricultural Sciences, Beijing, 100193 China; 2 Guangdong Laboratory of Lingnan Modern Agriculture, Shenzhen; Genome Analysis Laboratory of the Ministry of Agriculture; Agricultural Genomics Institute at Shenzhen, Chinese Academy of Agricultural Sciences, Shenzhen, 518120 China; 3 Functional and Evolutionary Entomology, Gembloux Agro-Bio Tech, University of Liège, Gembloux, 5030 Belgium; 4 Ministry of Agriculture Key Lab of Molecular Biology of Crop Pathogens and Insects, Institute of Insect Sciences, Zhejiang University, Hangzhou, China

**Keywords:** Agricultural genetics, Genome

## Abstract

The spotted alfalfa aphid (SAA, *Therioaphis trifolii*) (Hemiptera: Aphididae) is a destructive pest of cultivated alfalfa (*Medicago sativa* L.) that leads to large financial losses in the livestock industry around the world. Here, we present a chromosome-scale genome assembly of *T. trifolii*, the first genome assembly for the aphid subfamily Calaphidinae. Using PacBio long-read sequencing, Illumina sequencing, and Hi-C scaffolding techniques, a 541.26 Mb genome was generated, with 90.01% of the assembly anchored into eight scaffolds, and the contig and scaffold N50 are 2.54 Mb and 44.77 Mb, respectively. BUSCO assessment showed a completeness score of 96.6%. A total of 13,684 protein-coding genes were predicted. The high-quality genome assembly of *T. trifolii* not only provides a genomic resource for the more complete analysis of aphid evolution, but also provides insights into the ecological adaptation and insecticide resistance of *T. trifolii*.

## Background & Summary

Alfalfa (*Medicago sativa* L.), also called lucerne, is one of the world’s most important cultivated fodder plants. It is cultivated in at least 80 countries, and because it is an abundant and stable source of nutrients, it has become the backbone of the global livestock industry^1–3^. The spotted alfalfa aphid (SAA), *Therioaphis trifolii*, is one of the most serious insect pests of legumes, mainly causing the wide-scale destruction of alfalfa crops^4^. *T. trifolii* was first recorded in New Mexico in the United States of America^5^, and it also occurs in many regions of Australia, China, Europe, India, the Middle East, and the Mediterranean^5–7^. SAA damages its host plants by extracting nutrients from the leaves and phloem, and also by transmitting plant-pathogenic viruses, such as alfalfa mosaic virus and bean yellow mosaic virus^8^, thereby severely restricting the growth of plants and causing devastating losses in alfalfa production^9,10^.

The intensive use of chemical insecticides is the primary means of controlling aphids on many crops; however, this approach has become more challenging because aphids possess a great capacity to overcome multiple insecticides through the evolution of resistance^11–13^. Detoxifying enzymes contribute considerably to the development of insecticide resistance in aphids. For example, the peach potato aphid *Myzus persicae* is able to generate resistance to sulfoxaflor via overexpression of many detoxification-related enzymes, including UDP-glucuronosyltransferase (UGT) and cytochrome P450 (CYP) enzymes^14^, and *Aphis gossypii* overcomes sulfoxaflor through the up-regulation of ATP-binding cassette (ABC) transporter expression^15^. One practical and sustainable strategy to reduce insecticide applications is the cultivation of aphid-resistant plants^16^. Many studies have mined for specific genes that can generate durable genetic resistance to *T. trifolii* in plants^17–19^; however, the molecular mechanisms by which *T. trifolii* responds to aphid-resistant alfalfa plants still remain unclear. Studies on other aphids have revealed that many digestive proteases may be involved in overcoming the defenses of aphid-resistant plants. For example, significant changes in the expression levels of various digestion-related genes, such as serine proteases (SPs) and carboxypeptidases (CPs), have been detected in *Aphis glycines* after feeding on resistant soybean^20^. The availability of a high-quality genome sequence will be considerably beneficial for gaining an improved understanding of the molecular mechanisms underlying SAA resistance to pesticides and aphid-resistant alfalfa.

Taking advantage of the feasibility of inexpensive sequencing, researchers have sequenced the genomes of many aphids^21–26^, but the number of available genomes is still limited compared with the number of recorded aphids (more than 5000 species)^27^. In addition, most of the sequenced aphids belong to the subfamily Aphidinae, one large group consisting of various important pests, and only a few efforts have focused on other subfamilies^28,29^. The lack of genome sequences for other subfamilies has greatly limited our understanding of the genomic diversity and evolution of aphids. Calaphidinae is the second largest subfamily within the family Aphididae^30^; it consists of nearly 400 valid species, some of which are notorious pests damaging a distinctive range of host plants^31,32^. However, despite its importance, no reference genome is yet available for this group.

Here, we present a high-quality chromosome-level genome assembly of *T. trifolii*, generated using a combination of PacBio, Illumina, and chromatin conformation capture (Hi-C) techniques. Phylogenetic analysis was performed to determine the relationship of SAA with other members of the superfamily Aphidoidea. Moreover, annotation and comparative analyses of digestion- and detoxification-related gene families and genome synteny analyses were carried out between *T. trifolii* and other representative aphid species. Our study provides the first genome assembly for a Calaphidinae aphid, which will facilitate studies on the genome evolution of aphids and also significantly benefit efforts to control this important alfalfa pest.

## Methods

### Sample preparation and genomic sequencing

The *T. trifolii* colony was collected from the alfalfa fields at the Langfang Experimental Station of the Chinese Academy of Agricultural Sciences and reared on alfalfa (*Medicago sativa*) in natural light in a greenhouse maintained at 20 ± 2°C and relative humidity of 75%. We aimed to create a colony consisting entirely of asexual females, so we carefully selected a single female from the original population to establish a new colony. From this colony, we selected one offspring to generate the next colony, and we repeated this process until we obtained the fifth aphid colony, which comprised solely and steadily asexual females. This pure parthenogenetic colony was used as the sample for all the genome sequencing experiments.

For PacBio sequencing, DNA was extracted from about 200 individuals, consisting of wingless parthenogenetic female adults and nymphs. Two single-end 20-kb libraries were constructed with the PacBio SMRT (Single-Molecule Real-Time) sequencing (Pacific Biosciences). Raw reads were generated from one cell sequenced on the PacBio Sequel II platform. After quality control filtering, 118.55 Gb (~220 × coverage) of SMRT PacBio sequences were obtained, with a mean read length of 14.40 kb (N50 = 21.04 kb). For Illumina sequencing, about 200 wingless parthenogenetic female adults and nymphs were used for DNA extraction, and the library (400-bp inserts) was constructed using standard Illumina protocols and sequenced on the Illumina HiSeq X Ten platform, generating 33.73 Gb of data with 150 bp paired-end reads. To further assemble the contigs into chromosomes, we generated a Hi-C library using protocols described in a previous study^33^. Fresh tissues from about 150 individual samples (including adults and nymphs) were crosslinked with paraformaldehyde to obtain the interacting DNA segments. The cross-linked sample was digested with DpnII, and biotinylated nucleotides were used to label the ends of the restriction fragments. The library was quantified and sequenced on the Illumina Novaseq/MGI-2000 platform, and ~49.21 Gb of data with 150 bp paired-end sequencing raw reads were generated.

### RNA sequencing

Total RNA was extracted from 100 adult parthenogenetic female adults using TRIzol reagent (Invitrogen, Carlsbad, CA, USA)^34^ and dissolved in RNase-free water. The integrity of the RNA was assessed by 1% agarose gel electrophoresis. RNA purity and concentration were assessed using a Nanodrop ND-2000 spectrophotometer (ThermoFisher, USA). The qualified RNA was used for constructing cDNA libraries. Raw sequencing data were generated using an Illumina NovaSeq 6000 platform (Illumina, San Diego, CA, USA) with the 200 bp paired-end strategy. A total of 93,816,908 clean reads were generated with a Q30 rate exceeding 90%.

### Genome assembly

The quality control of raw Illumina reads was carried out using FASTP v0.20.0^35^. Clean reads were used to construct a 17-mer frequency distribution map using JELLYFISH v2.3.0^36^. The genome size of *T. trifolii* was estimated to be 542.4 Mb based on k-mer analysis.

For contig assembly, we first used FALCON v1.8.7 (reads_cutoff: 1k,seed_cutoff: 33k)^37^ for the error correction of PacBio reads. The corrected reads were assembled into the preliminary genome assembly using SMARTDENOVO v1.0 with parameters -J 3000 and -k 19^38^. To correct errors generated during the assembly process, PacBio reads were mapped to the genome using BLASR v5.1^39^, and ARROW v2.2.2 was used for one round of genome polishing with default parameters. Illumina reads were also mapped to the assembly using BWA v0.7.12^40^, and then four iterations of contig polishing were carried out using NEXTPOLISH v1.0.5 with default parameters^41^. A contig-level assembly with a total length of 541.26 Mb was generated, which is comparable to the estimated genome size, and the contig N50 length was 2.54 Mb (Table 1).

**Table 1:** Major indicators of the *Therioaphis trifolii* genome.

Features	Statistics
Estimated genome size (bp)	542,395,090
Assembly size (bp)	541,263,359
Contigs N50 (bp)	2,544,558
Scaffolds number	575
Scaffolds N50 (bp)	44,770,504
BUSCO genes	C: 96.6% [S: 93.5%, D: 3.1%], F: 0.7%
Number of protein-coding genes	13,684

### Hi-C scaffolding

Low-quality raw reads (quality score < 20 and shorter than 30 bp) and adaptors were removed using FASTP v0.20.0, then the clean reads were mapped to the contig assembly using BOWTIE2 v2.3.2 (-end-to-end–very-sensitive -L 30)^42^. HI-C PRO v2.8.1^43^ with default parameters was used to identify valid interaction paired reads and to filter out reads with multiple hits and singleton reads. LACHESIS^44^ was used to cluster, order, and orient the contigs with parameters CLUSTER MIN RE SITES = 100; CLUSTER MAX LINK DENSITY = 2.5; CLUSTER NONINFORMATIVE RATIO = 1.4; ORDER MIN N RES IN TRUNK = 60; ORDER MIN N RES IN SHREDS = 60.

**Fig. 1:**
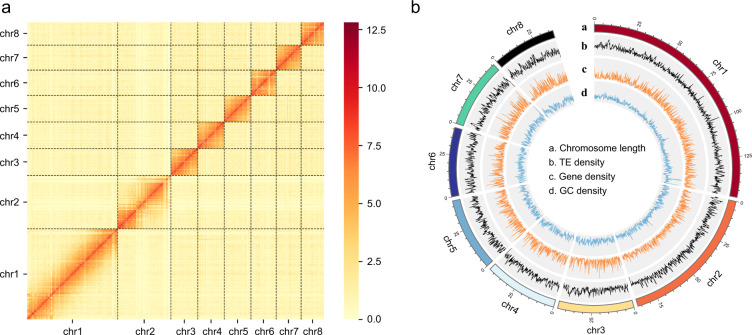
Heatmap of genome-wide Hi-C data and circular representation of the chromosomes of *Therioaphis trifolii*. (**a**) The heatmap of chromosome interactions in *T. trifolii*. The frequency of Hi-C interaction links is represented by colours, which ranges from yellow (low) to red (high). (**b**) Circos plot of distribution of the genomic elements in *T. trifolii*. The tracks indicate **a**) length of the chromosome, **b**) distribution of transposable element (TE) density ranges from 11 to 502, **c**) gene density ranges from 0 to 17, and **d**) GC density ranges from 22 to 62. The densities of TEs, genes, and GC were calculated in 100 kb windows.

As a result, Hi-C data were combined with the contig-level assembly to generate a chromosome-level assembly comprising eight large scaffolds (Fig. 1a), which corresponds to the previously reported haploid chromosome number for this species^45^. Around 90.07% of the contigs were anchored onto chromosomes, resulting in a scaffold N50 length of 44.77 Mb (Table 1). The longest chromosome was 149.16 Mb while the shortest was 37.54 Mb (Fig. 1b).

### Repeat annotation

TANDEM REPEAT FINDER v4.07b (parameters: 2 7 7 80 10 50 500 -f -d -h -r)^46^ was used to identify all tandem repeat elements. Transposable elements (TEs) were identified using a combination of two methods. First, a de novo repeat library was generated using REPEATMODELER v1.0.11 and MITE-hunter^47^ with default parameters. This library was searched against the Repbase^48^ to classify repeat families using REPEATMASKER v1.331, and then merged with Repbase to generate the final repeat sequence library. Next, REPEATMASKER v1.331 was used to predict TEs based on the final TE library. The result showed that repeat sequences make up 36.86% of the genome, most of which are TEs (33.31%) (Table 2).

**Table 2:** Statistics of the transposable elements in *Therioaphis trifolii* genome.

Repeat types	Number of elements	Length occupied (bp)	Percentage of sequence
SINE	5,290	506,439	0.09%
LINE	135,921	27,613,234	5.10%
LTR	73,841	14,343,500	2.65%
MITE	53,954	13,738,880	2.54%
DNA	716,470	121,720,828	22.49%
RC	18,203	2,394,844	0.44%
Unknown	45,373	7,137,015	1.32%
Total base masked	1,236,028	199,531,663	36.86%

### Protein coding gene prediction and functional annotation

Gene model prediction from the TE soft-masked *T. trifolii* genome was performed using multiple approaches, namely transcriptome-based prediction, ab initio prediction, and homology-based gene prediction. For transcriptome-based analysis, clean reads were aligned to the genome assembly using STAR v2.7.3a^49^ with the default parameters. Next, STRINGTIE v1.3.4d^50^ was used to obtain transcript locations, and open reading frames of the transcripts were predicted using PASA v2.3.3^51^. For de novo gene model prediction, the transcript set generated by PASA was utilized by GENEMARK-ST v5.1^52^ for self-training. The training set was applied to AUGUSTUS v3.3.1^53^ for gene model prediction. For the homology-based gene modeling process, protein sets from three aphids with high-quality genome assemblies (consisting of *A. pisum*, *R. maidis* and *S. miscanthi*) were aligned to the genome assembly via GEMOMA v1.6.1^54^. Finally, we combined the results from the three gene prediction approaches to create a consensus gene model set using EVIDENCEMODELER v1.1.1 (–segmentSize 1000000–overlapSize 100000)^51^. As a result, 13,684 protein-coding gene models were generated, with an average gene length of 15 kb, average coding sequence length of 1.5 kb, and average exon number of 7.1.

For gene functional annotation, protein sequences encoded by the predicted gene models were aligned to the non-redundant (nr), SWISS-PROT, Kyoto Encyclopedia of Genes and Genomes (KEGG) (Kanehisa & Goto, 2000), and eukaryotic orthologous groups (KOG) databases (Galperin *et al*., 2015) using BLASTP v2.7.1 with a cutoff of 1e-5. We also used INTERPROSCAN v5.32-71.0^55^ to obtain gene ontology (GO) annotations for the proteins.

### Phylogenetic and comparative genomic analyses

**Fig. 2:**
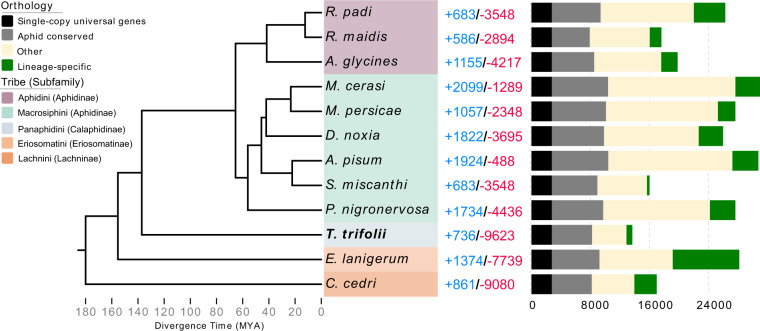
Phylogeny and orthology analyses between *Therioaphis trifolii* and other aphid species. The phylogenetic tree was constructed based on 2,758 single-copy orthogroups obtained from the genomes of all tested aphids. The greenhouse whitefly *Trialeurodes vaporariorum* (not shown) was selected as the outgroup. Aphid species are colored according to their tribe. Numbers of expanded (blue) and contracted (red) gene families are presented alongside the species and nodes. The bar chart shows the comparison of orthologs between 12 aphids. ‘Single-copy universal’ refers to a single-copy ortholog that is present in all the aphids. ‘Aphid conserved’ refers to genes that can be detected in at least 11 aphid genomes. ‘Lineage-specific’ refers to  genes that do not have an ortholog in any other aphid. ‘Other’ indicates orthologs are found in some of the aphids (e.g., in 1 to 10 aphids).

The longest predicted protein sequences of 12 aphid genomes, namely *Aphis glycines*^56^, *Acyrthosiphon pisum*^22^, *Cinara cedri*^28^, *Diuraphis noxia*^57^, *Eriosoma lanigerum*^29^, *Myzus cerasi*^58^, *Myzus perisicae*^22^, *Pentalonia nigronervosa*^59^, *Rhopalosiphum maidis*^24^, *Rhopalosiphum padi*^58^, *Sitobion miscanthi*^23^, and *T. trifolii*, and the greenhouse whitefly *Trialeurodes vaporariorum*^60^, which was used as an outgroup, were utilized for identifying orthologous groups among aphids using ORTHOFINDER v2.4.0^61^. A total of 2758 single-copy orthogroups were identified and used to generate a concatenated alignment for inferring phylogenetic relationships. The species tree of the 12 aphids was also inferred using ORTHOFINDER^62^ and rooted by STRIDE^63^. Divergence times among aphids were calculated by R8S^64^ based on divergence information extracted from TimeTree (http://www.timetree.org/): *A. pisum* vs *M. persicae* 42.5–48.0 million years ago (mya) (Fig. 2). We also used CAFE v4.2.1^65^ to analyze the expansion and contraction of gene families in all 12 tested aphid lineages. The results from the phylogenetic tree with divergence times were used as inputs (Fig. 2).

### Analysis of detoxification- and digestion- related genes

**Fig. 3:**
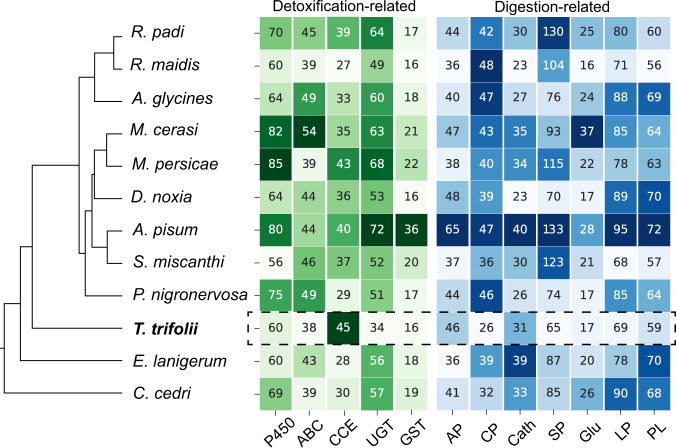
Detoxification- and digestion-related gene families in *Therioaphis trifolii*. The maximum likelihood phylogeny, based on a concatenated alignment of 2,758 single-copy orthogroups, illustrates the phylogenomic relationship between *T. trifolii* and other aphids. Numbers in the heatmaps indicate the size of the corresponding gene family in aphid species. Detoxification-related and digestion-related gene families are labeled with a green and blue shade, respectively.The darker shade of color indicates a higher number of identified genes.

The amino acid sequences of each aphid were searched against the NR and SWISS-PROT databases using DIAMOND v0.9.21^66^ with an e-value cut-off of 1e-5, and INTERPROSCAN was used to predict functional domains in each sequence. Genes encoding the best scoring protein hits of digestion-related and detoxification-related enzymes were annotated according to the best hit (Fig. 3).

### Synteny analysis

**Fig. 4:**
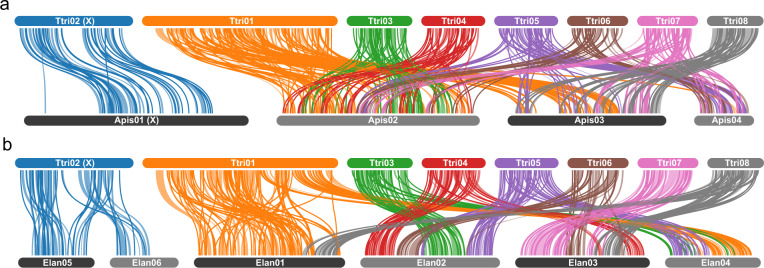
Genome synteny between (**a**) *Therioaphis trifolii* (Calaphidinae) and *Acythosiphon pisum* (Aphidinae) and (**b**) *T. trifolii* and *Eriosoma lanigerum* (Eriosomatinae). Links indicate the edges of syntenic blocks of gene pairs identified by synteny analyses and are shown in the same color as that of the chromosome ID of *T. trifolii*. Ttri indicates *T. trifolii*, Apis indicates *A. pisum*, and Elan indicates *E. lanigerum*.

The synteny analyses were carried out between the chromosome-level genome assemblies of *T. trifolii*, *A. pisum* (JIC1 v1), and *E. lanigerum*. To obtain syntenic blocks, we uploaded the official gene sets to the ORTHOVENN2 server^67^. The 1:1 single-copy ortholog pairs from each comparison (*T. trifolii* vs *A. pisum* and *T. trifolii* vs *E. lanigerum*) were identified using the parameters e-value = 1e-5 and inflation value = 1.5. These gene pairs were selected for genome synteny analyses using MCSCANX v1.1^68^ with default parameters. SYNVISIO (https://synvisio.github.io) was used to visualize genome synteny (Fig. 4).

## Data Records

The genome sequencing, RNA sequencing reads data has been updated to the National Center for Biotechnology Information (NCBI) as a BioProject no. PRJNA804007. Pacbio, Hi-C, Illumina and transcriptome sequencing reads have been deposited in the Sequence Read Archive (SRA) databases with the accession number of SRP359015^69^. Genome assembly has been deposited at the NCBI under the accession number of JALBXZ000000000^70^, and can be download from National Genomic Data Center (NGDC) under accession number GWHBQDZ00000000.1. The annotated detoxification and digestion related genes among aphids have been uploaded to the NGDC under accession number OMIX002672. Gene sequences predicted from the genome assembly are also publicly available in NGDC, under the accession number OMIX002673. The statistics of RNA-Seq data have been deposited to the NGDC under accession number OMIX003518. All data in NGDC were related to the BioProject PRJCA014018. The TE library, sequences used for orthology analysis, the results of ORTHOFINDER and ORTHOVENN2 are available at Zenodo^71^.

## Technical Validation

The accuracy and completeness of the contig assembly were validated using four methods. First, clean Illumina reads were mapped to the contigs assembled by BWA v0.7.12, and the total mapped reads and mapping rate were calculated using SAMTOOLS v1.4^72^, resulting in a mapping rate of 99.40%. Second, clean reads from 4 whole-body transcriptomes were mapped onto the genome assembly, more than 97% of the RNA-Seq reads can be aligned to the coding regions of the genome assembly. Third, Benchmarking Universal Single-Copy Orthologs (BUSCO) v4.0.5^73^ was employed to assess the completeness of the genome assembly based on the insecta_odb10 database (-l insecta_odb10 -m genome), the BUSCO analysis indicated that 97.3% of gene orthologs were identified in *T. trifolii*, including complete and fragment scores of 96.6% and 0.7%, respectively. Finally, CEGMA v2^74^ with default parameters was used to validate the integrity of the core genes in the assembly, 242 core eukaryotic genes were assembled, among which 94.76% were complete.

To ensure the completeness of the annotated gene set, four validation methods were employed. Firstly, the annotation was subjected to BUSCO analysis using the insecta_odb10 database (-l insecta_odb10 -m prot). The results indicated that 95.98% of the conserved single copy ortholog genes, including 95.39% of complete genes and 0.59% of fragmented genes, were present in the annotated protein set. Secondly, gene expression analysis was conducted using RNA-Seq reads from four whole-body transcriptomes. The analysis revealed that 11,422 (83.47%) annotated genes were expressed in at least one transcriptomic sample, and 72.91%~74.32% of the RNA-seq reads could be assigned onto the coding region of the genome assembly. Thirdly, to assess the completeness of annotated gene structures, the length ratio of predicted proteins to their best hit in the proteomes of three high-quality aphid genome assemblies (*A. pisum*, *M. persicae*, *and R. maidis*) was analyzed. Predicted proteins with a ratio of 0.9–1.1 were considered high confidence predictions. The results showed that a large number of high confidence predictions were obtained, including 8,850 (64.7%), 8,876 (64.9%), and 8,611 (62.9%) from the *T. trifolii* versus *A. pisum*, *T. trifolii* versus *M. persicae*, and *T. trifolii* versus *R. maidis* comparisons, respectively. Finally, the predicted gene models were compared against several protein databases (nr, SWISS-PROT, GO, KOG, and KEGG). The results showed that 12,995 (94.96%) of the predicted gene models had significant homology to proteins in at least one of these databases.

## Data Availability

All software and pipelines used for data processing were executed according to the manuals and protocols of the bioinformatics software cited above, and the parameters are clearly described in the Methods section. If no detailed parameters are mentioned for a software, the default parameters were used. The version of the software has been described in Methods.
